# Books for Sick Men

**Published:** 1920-12-04

**Authors:** 


THE PUBLIC WELL-BEING—(*continued*).

### Books for Sick Men.

At a recent meeting held under the auspices of the League of Remembrance, some of the members gave an interesting account of their experiences as librarians in the war hospitals. During the war the British Red Cross Society supplied the hospitals with nearly 150,000 books and magazines. The requests of the sick and wounded for literature often took a very unusual form. One man found relief from boredom in a large railway guide, by means of which he took imaginary trips to different parts of the country, working out third-class return fares with great gusto; and another refused to be comforted until he had been provided with a Japanese grammar.

But I like best (writes an ex-Army officer) the reply of a lonely man, who, on being appealed to to make a choice of some kind, remarked fervently "I never read anything, and please God I never shall." That is more characteristic of wounded men in the war than most people would be inclined to think. I knew some who could not read, but a great many who were too lazy to read even the newspaper containing the result of the football matches they were specially interested in, but invariably persuaded a good-natured comrade to read the contents aloud to them. My experience of Thomas Atkin's literary longings in hospital is that, generally speaking, he liked nothing so much as a sentimental novel, reeking of sobs and sighs and kisses; and that he liked next books about sport, while travel books were usually in great request. Books about the war were less popular than technical treatises.

What I would recommend for the sick-bed of an ordinary reasonably well-educated man is a volume of light essays. The essay form makes just the right kind of mental demand on a convalescent person by attracting and holding his attention without exacting too much concentration or exerting emotions too fierce or painful. No man ought to complain of *ennui* with a volume of R.L.S. in his hand, and one of Lamb on the bed by his side. And there are twenty-five others I could name of more modern date, but I might be looked upon as a disguised agent for the publishers and authors. Emerging from a very serious illness I once conceived an absurd passion for poring over the profundities of Hoffding's "Outlines of Psychology." I read it from end to end; but it made me very irritable, and was the cause, so the nurse declared, of a bad attack of indigestion that left me more irritable still. But any medical practitioner will tell you that in nine cases out of ten there is no better medicine in the world than a cheerful book for the man who has just turned his back on death and is beginning to struggle back to health. The art of printing has been a useful handmaiden in more ways than one to the practice of medicine.

